# Relationship between dementia and ankylosing spondylitis: A nationwide, population-based, retrospective longitudinal cohort study

**DOI:** 10.1371/journal.pone.0210335

**Published:** 2019-01-31

**Authors:** Hae-Dong Jang, Jin-Sung Park, Dae Woong Kim, Kyungdo Han, Byung-Joon Shin, Jae Chul Lee, Sung-Woo Choi, Seung-Woo Suh, Jae-Hyuk Yang, Si-Young Park, Whi Je Cho, Jae-Young Hong

**Affiliations:** 1 Department of Orthopaedic Surgery, Soonchunhyang University Hospital, Bucheon-si, Gyeonggi-do, South Korea; 2 Department of Orthopedics, Korea University Hospital, Ansan, Danwon-gu, Ansan-si, Gyeonggi-do, South Korea; 3 Department of Orthopaedic Surgery, Soonchunhyang University Hospital, Yongsan-gu, Seoul, South Korea; 4 Department of Biostatistics, College of Medicine, Catholic University, Jongno-gu, Seoul, South Korea; 5 Scoliosis Research Institute, Department of Orthopedics, Korea University Medical College, Guro Hospital, Guro-gu, Seoul, South Korea; 6 Department of Orthopaedic Surgery, Korea University, College of Medicine, Anam Hospital, Seongbuk-gu, Seoul, South Korea; Universiti Sains Malaysia, MALAYSIA

## Abstract

Among a variety of comorbidities of ankylosing spondylitis (AS), the association between dementia and AS by using an extensive dataset from the Korean National Health Insurance System was evaluated in this study. We extracted 15,547 newly diagnosed AS subjects among the entire Korean population and excluded wash-out patients (n = 162) and patients that were inappropriate for cohort match (n = 1192). Finally, 14,193 subjects were chosen as the AS group, and through 1:5 age- and sex-stratified matching, 70,965 subjects were chosen as the control group. We evaluated patient demographics, household incomes, and comorbidities, including hypertension, diabetes, and dyslipidemia. The prevalence of overall dementia (1.37%) and Alzheimer’s dementia (AD) (0.99%) in the AS group was significantly higher than in the control group (0.87% and 0.63%), respectively. The adjusted hazard ratio of the AS group for overall dementia (1.758) and AD (1.782) showed statistical significance also. On the other hand, the prevalence of vascular dementia did not differ significantly between the two groups. Subgroup analyses revealed the following risk factors for dementia in the AS group: male gender, greater than 65 years in age, fair income (household income greater than 20% of the median), urban residency, no diabetes, and no hypertension. From the nationwide, population-based, retrospective, longitudinal cohort study, AS patients showed a significantly higher prevalence of overall dementia and Alzheimer’s dementia. Comprehensive patient assessment using our subgroup analysis could help to prevent dementia in patients suffering from AS.

## Introduction

Ankylosing spondylitis (AS) is a chronic inflammatory disease caused by enthesopathy and arthritis of the axial skeleton. The condition leads to frequent back pain and stiffness of the affected joints.[1–3] Because there is no cure for the disease itself and because onset typically happens early in young adults who are socially active, AS patients obviously suffer from decreased quality of life (QOL) with long-lasting emotional and functional impairments.[4–12] These disease characteristics also affect the disease comorbidities: risks of major degenerative diseases—including cardiovascular or cerebrovascular problems—and mental disorders in AS are significantly higher than in the general population, as reported in several published papers.[4,5,7,9–15] Especially among psychological abnormalities, the association between AS and depression, anxiety, and sleep disturbances have been well studied.[5,6,8–10,12] Currently the same cannot be said for the measurement of dementia (Alzheimer’s or vascular), despite their clinical importance. Dementia is known as the representative elderly mental illness, with a high prevalence (10% of men greater than 65 years worldwide) that increases the socio-economic burden in recently aging societies worldwide.[16] In 2012, the World Health Organization announced that dementia is one of the highest priority public health issues globally. [16] According to the World Alzheimer's Disease Report 2015, the number of dementia patients worldwide is estimated at 46 million, with 74 million cases forecast by 2030 and 131 million by 2050. In addition, the prevalence of dementia rapidly increases with aging, and the prevalence increases twice every five years after age 65. [17]

Based on the results of the international survey (mean prevalence of AS per 10,000 people; 16.7 in Asia, 23.8 in Europe, and 31.9 in North America), AS is not a common disease; therefore, large-scale research, such as nationwide, population-based studies, is essential.[16,17] Furthermore, since an evaluation for the trend and association between two chronic diseases (AS and dementia) needs statistical adjustment for multiple covariant, the extensive dataset surveyed in Korea by the National Health Insurance System (NHIS) could ensure a high-quality cohort study. Most studies dealing with comorbidities of AS were based on cross-sectional data, and to date, there has been no study based on nationwide, longitudinal follow-up data.[4,5,7,8,14]

We hypothesized that patients with AS may have a higher risk of developing dementia; therefore, we conducted a nationwide, population-based, retrospective cohort analysis in the Korean NHIS (K-NHIS) claim database to investigate a possible association between these two illnesses.

## Materials and methods

### Data source

Data were obtained from the K-NHIS database surveyed from 2010 to 2014. The K-NHIS program covers approximately 97% of people in the entire nation for any medical procedure, with the exception of cosmetic surgery (procedures that patients wanted rather than needed) and services for a motor vehicle collision. The remaining 3% (low-income people) were included in the Medical Aid program. The K-NHIS claim database includes extensive information about diagnoses (as defined by the 10th revised codes of the International Classification of Diseases [ICD-10]), demographics, medical service (treatment and procedures) and costs of both inpatients (hospital admission or hospitalization) and outpatients (ambulatory care) submitted from all clinics and hospitals. However, the K-NHIS claim database did not include the detailed clinical data including laboratory results (erythrocyte sedimentation rate or C-reactive protein), radiological findings, or clinical scores (Bath Ankylosing Spondylitis Disease Activity Index or Bath Ankylosing Spondylitis Functional Index). Therefore, data or information about the status of AS inflammation did not evaluate in the present study.

### Study design and subjects

To identify patients with AS (the AS group), we searched for claims listing an ICD-10 code of M45 and Reduction of Medical Expenses for Rare Complaints Code of V14.0 based on the diagnostic criteria of AS between January 1, 2010, and December 31, 2014. To investigate the risk of dementia in patients with AS and compare the variables of case and control groups, we established an age- and sex-matched cohort control group. Dementia was defined as existing if anti-dementia drugs were prescribed at least two times and the codes for Alzheimer’s disease (ICD-10 F00 or G30), vascular dementia (ICD-10 F01.0, F01.1, F01.2, F01.3, F01.8, or F01.9), or other dementia (ICD-10 F02, F03, G23.1, G31.0, G31.1, G31.82, G31.83, G31.88, or F10.7) were assigned. Anti-dementia drugs, belonging to four classes, included rivastigmine, galantamine, memantine, and donepezil hydrochloride. The detailed diagnosis of dementia was defined as the first prescription of anti-dementia drugs since a diagnosis. When there were both Alzheimer’s disease (AD) and vascular dementia (two codes each), the final diagnosis was defined as vascular dementia (VD). If there was neither AD nor VD, the diagnosis was defined as other dementia. Subjects in this study were followed from the first occurrence of dementia to death or the end of follow-up (whichever occurred first). We evaluated the patient demographics (sex, age, and region of residence), household income, and comorbidities including hypertension, diabetes, and dyslipidemia.

### Establishment of the study cohort

**Fig 1:**
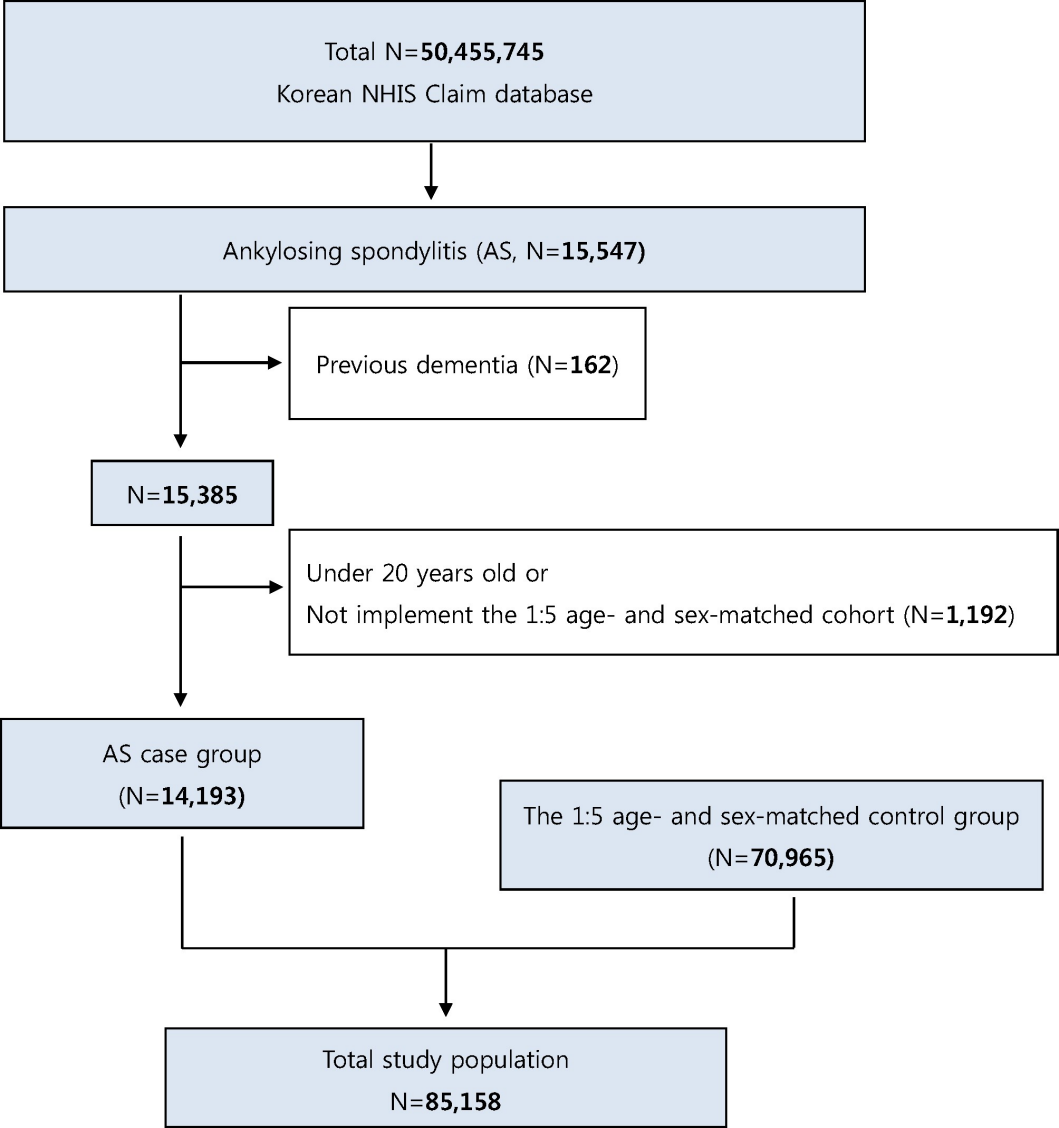
Flowchart of the inclusion and exclusion for subjects based on the dataset surveyed in Korea by the National Health Insurance System (NHIS).

We extracted 15,547 newly diagnosed AS subjects from January 1, 2010, to December 31, 2014, from among the entire population of Korea. At first, 162 subjects who were diagnosed with dementia at least once during the period from January 1, 2002 (start of K-NHIS database) until any date before the first diagnosis date of AS were excluded as adequate wash-out patients (Fig 1). Afterward, 1192 subjects were excluded; subjects younger than 20 years old or who could not be fit the 1:5 age- and sex-matched control cohort. Finally, 14,193 subjects were chosen as the AS case group. Through the 1:5 age- and sex-stratified matching without replacements performed by a greedy digit match algorithm, 70,965 subjects were decided as the control group. In all, 85,158 subjects including case (n = 14,193) and control (n = 70,965) groups were used in the present study (Fig 1).

### Statistical analysis

Data are presented as a mean ± standard error (SE) or as percentages with SE. In order to compare the variables of two groups, Students t-test and chi-square test were used. The Kaplan-Meier method was used to estimate the dementia-free survival probabilities of the two groups. Differences in disease-free survival between the two groups were tested using the Wilcoxon’s log-rank test. To evaluate the association between AS and dementia, multivariable analyses were conducted using the Cox proportional hazard regression model. In the adjusted analyses, age, sex, income, hypertension, diabetes, and dyslipidemia were used as confounding variables. Parameters with a P-value less than 0.15 for the *t*-tests and chi-square tests were selected for multivariable analyses. In order to estimate the effect of AS on the subsequent occurrence of each event (dementia), in each subgroup analysis according to the confounding variables, two Cox proportional hazard regression models were performed. Model 1 estimated the dementia incidence after adjustment for sex and age. Hazard ratios were calculated after adjusting for sex, age, income, and other comorbidities in Model 2. Statistical analysis was performed with SAS survey procedures (version 9.3; SAS Institute, Cary, NC, USA). P-values less than 0.05 were considered statistically significant.

### Ethics statement

The study protocol was approved by the NHIS institutional review board (number: NHIS-2018-1-115). Informed consent was exempted by the board because the data used consisted of de-identified secondary data released for research purposes. The present study was approved by the institutional review boards at Korea University Hospital, Ansan (IRB No. 2018AS0002). All methods were performed in accordance with the relevant guidelines and regulations.

## Results

### Cohort characteristics

A total of 14,193 patients with AS (71.75% men) and 70,965 control subjects without AS were included in our study sample. Comparisons of the demographic and clinical variables between the patients with AS and control subjects are presented in Table 1. The mean age of the patients was 41.76 years, and by age subgroups, the under-40-year-old age group was most frequently greater than half of the patients, and the 40–64-year-old group was approximately 40%, and the greater-than-65-year-old group was less than 10%. Because of the 1:5 sex-stratified matching, the proportion of sex, mean age, and proportion of the age subgroups (less than 40, 40 to 65, greater than 65 years old) were exactly the same in the two groups (Table 1). In the AS group, the proportion of low household income, urban resident, and comorbidities (diabetes, hypertension, and dyslipidemia) were significantly higher than in the control group (p<0.0001). During the follow-up period, the prevalence of newly diagnosed dementia was 1.37% (n = 195) in the AS group and 0.87% (n = 615) in the control group. The proportion of AD in the AS group was significantly higher than the control group (p<0.0001). On the other hand, there were no significant differences in the proportion of VD between the two groups (p = 0.2515). Mean follow-up durations for dementia in the AS and control subjects were 3.47 and 3.43 years, respectively (p = 0.0131).

**Table 1:** Demographics and clinical characteristics of the patients with ankylosing spondylitis and control group.

Variables	AS (N = 14,193)	Control (N = 70,965)	*p*
**Demographics**			
Sex (Male)	10183 (71.75%)	50915 (71.75%)	1
Age (yr.)	41.76 ± 15.13	41.76 ± 15.13	1
Distribution of age			
< 40 yr.	7201 (50.74%)	36005 (50.74%)	1
40–64 yr.	5660 (39.88%)	28300 (39.88%)	1
≥ 65 yr.	1332 (9.38%)	6660 (9.38%)	1
Low income	3513 (24.75%)	15530 (21.88%)	**< .0001**
Place of residence (Urban)	6797 (48.56%)	32712 (46.24%)	**< .0001**
Comorbidities			
Diabetes	855 (6.02%)	3611 (5.09%)	**< .0001**
Hypertension	2386 (16.81%)	8901 (12.54%)	**< .0001**
Dyslipidemia	1561 (11%)	5543 (7.81%)	**< .0001**
Depression	1470 (10.36%)	2392 (3.37%)	**< .0001**
**Newly diagnosed dementia**			
Prevalence			
Overall dementia	195 (1.37%)	615 (0.87%)	**< .0001**
Alzheimer’s dementia	141 (0.99%)	444 (0.63%)	**< .0001**
Vascular dementia	23 (0.16%)	88 (0.12%)	0.2515
Follow-up duration of dementia (yr.)	3.43 ± 1.47	3.47 ± 1.46	**0.0131**

Mean ± standard deviation. Bold style indicates statistical significance (< 0.05). AS: ankylosing spondylitis; yr: years; Low income; household income less than 20 percent of the median

### The risk for Dementia (Vascular and Alzheimer’s) in the AS and control groups

**Fig 2:**
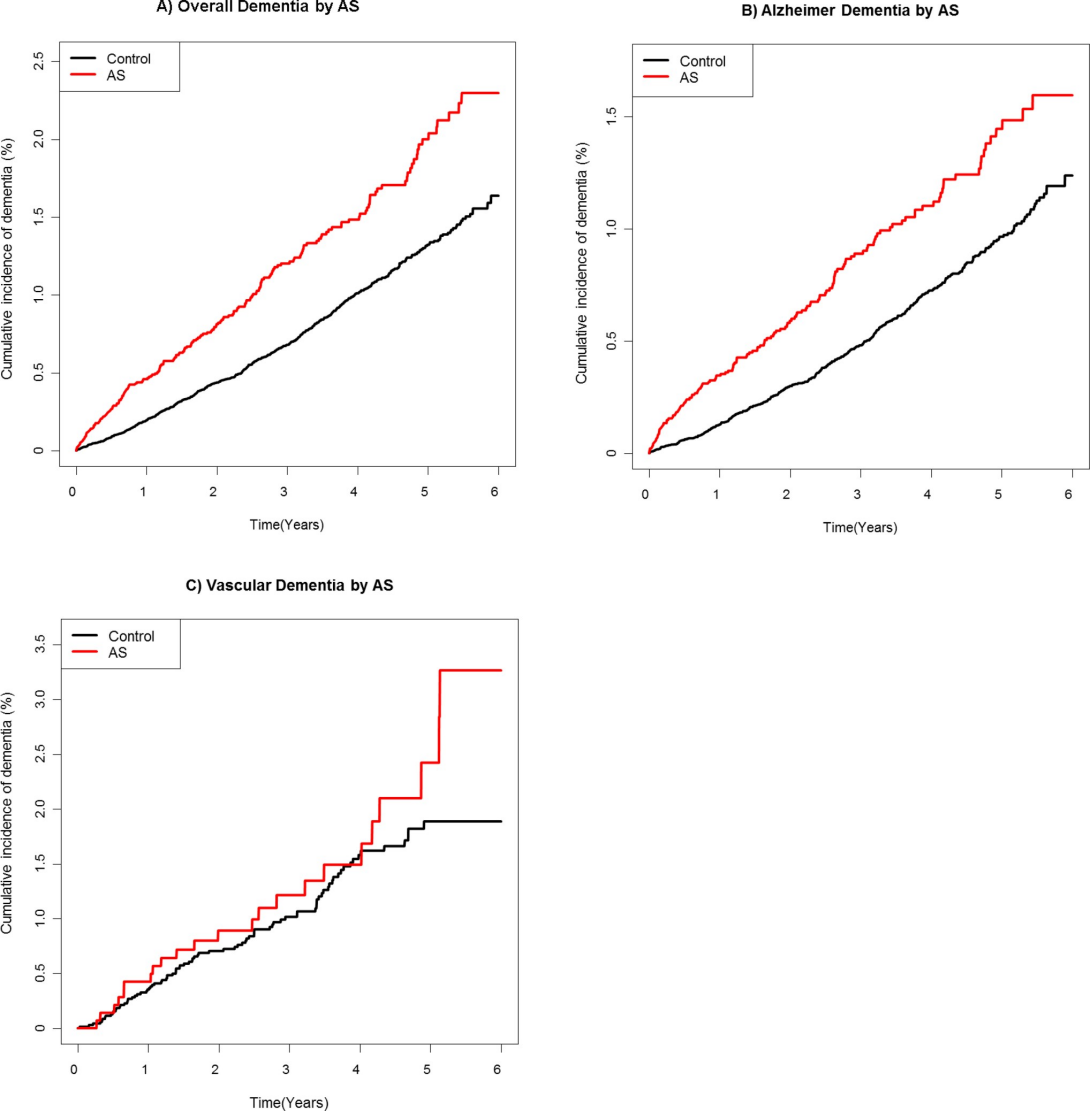
Comparison of the cumulative incidence of dementia (overall, Alzheimer’s, and vascular) in the AS and control groups. The Kaplan-Meier curves with cumulative hazards showed the significantly higher incidence of overall dementia (A) and Alzheimer’s dementia (B) in the AS group compared to the control group (p < 0.001). The cumulative incidence of VD was not statistically different between the two groups (C).

A Cox proportional hazards regression analysis based on the statistical adjustment (Model 1; age and sex, Model 2; age, sex, household income, diabetes, hypertension, dyslipidemia, and depression) was conducted to calculate the hazard ratio (HR) of the newly diagnosed dementia in patients with AS compared with matched controls (Table 2). The results indicated that patients with AS exhibited a markedly higher risk of developing a subsequent overall dementia (adjusted HR 1.758, 95% confidence interval (CI) 1.496–2.065 in Model 1 and adjusted HR 1.499, 95% CI 1.270–1.769 in Model 2) and AD (adjusted HR 1.782, 95% CI 1.474–2.154 in Model 1 and adjusted HR 1.554, 95% CI 1.278–1.888 in Model 2). On the other hand, VD showed adjusted HR 1.409 in Model 1 and 1.094 in Model 2 without significant 95% CI. After adjustment for the baseline variables, the Kaplan-Meier (KM) curves with cumulative hazards showed a significantly higher incidence of overall dementia and AD in the AS group than in the control group (p-value for log-rank test < 0.001) (Fig 2). Moreover, the gap difference between two KM curves of the AS and control groups increases with time in both overall dementia and AD comparison graphs (Fig 2A and 2B). The cumulative incidence of VD was not statistically different between the two groups (Fig 2C) (p = 0.2341).

**Table 2:** Calculated hazard ratios of dementia (Alzheimer’s and vascular) in the AS and control groups.

Type	AS	Event (N) (dementia)	Total follow-up duration (PY)	Incidence rate (per 1,000 PY)	Hazard ratio (95% CI)	
					Model1	Model2
**Overall** **Dementia**	**Control** **(N = 70965)**	615	245904	2.501	1	1
	**AS (N = 14193)**	195	48708.6	4.003	1.758 (1.496–2.065)	1.499 (1.270–1.769)
**Alzheimer’s Dementia**	**Control**	444	245904	1.806	1	1
	**AS**	141	48708.6	2.895	1.782 (1.474–2.154)	1.554 (1.278–1.888)
**Vascular Dementia**	**Control**	88	245904	0.358	1	1
	**AS**	23	48708.6	0.472	1.409 (0.89–2.23)	1.094 (0.683–1.752)

Incidence rate = event (dementia) / total follow-up duration. Model 1: adjusted for age, sex. Model 2: adjusted for age, sex, household income, diabetes, hypertension, dyslipidemia, and depression. AS: ankylosing spondylitis; PY: person-year; CI: confidence interval

### Subgroup analyses of risk for dementia in the AS group

**Fig 3:**
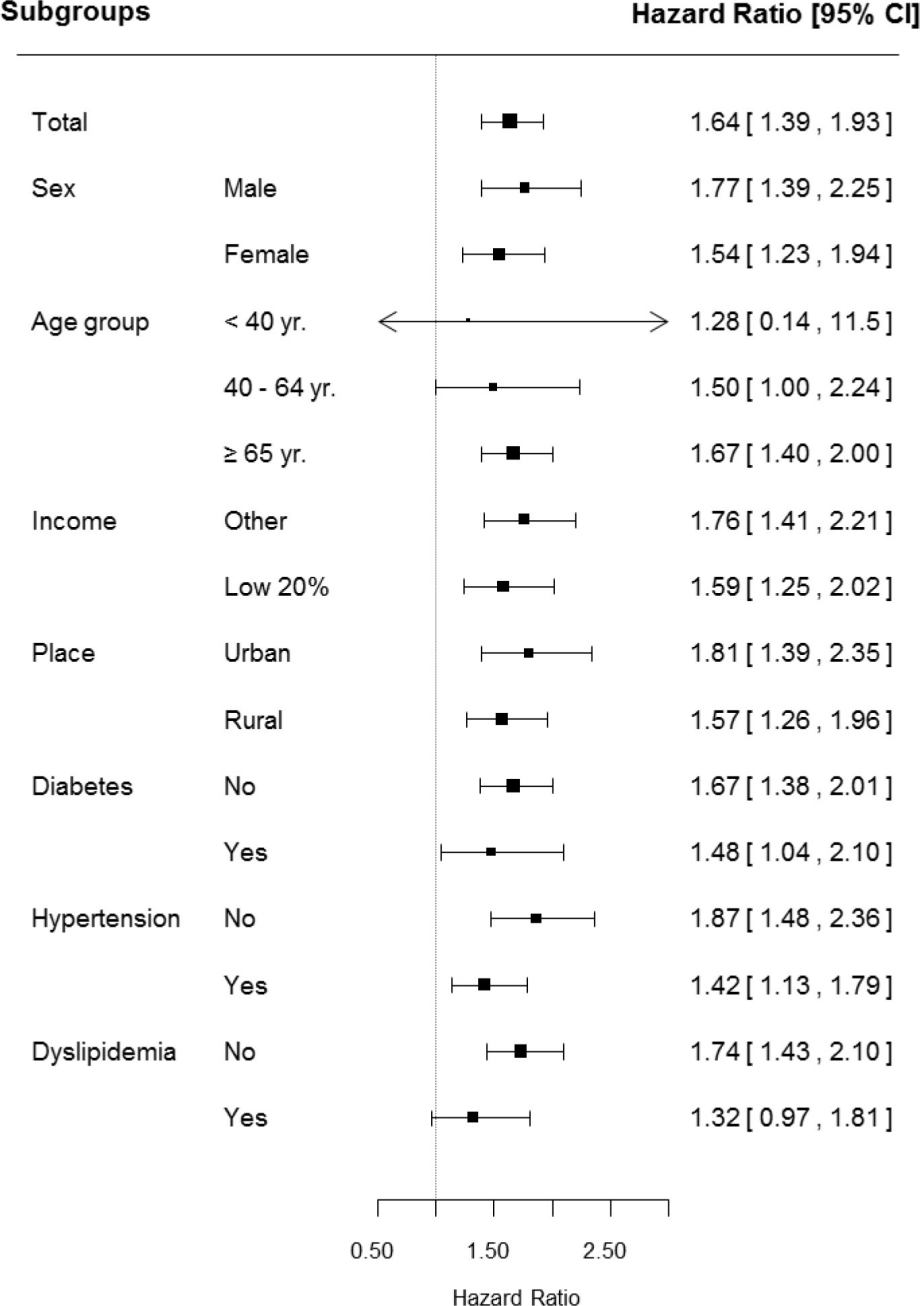
Subgroup analyses of risk for dementia in the AS group.

In the gender subgroup, male AS patients showed significantly higher HR (1.77) than patients in the female subgroup (1.54) with statistical significance (Fig 3). In age subgroups, the under-40-year-old group showed HR 1.28 for dementia incidence in AS patients, however, there was no statistical significance (95% CI, 0.14–11.5). On the other hand, in both the 40-to-64-year-old group and the greater-than-65-year-old group, a significant increase in dementia was observed. In the subgroup analysis, subgroups that showed significantly higher HR for dementia in AS were those with subjects with fair income (household income greater than 20% of the median) (HR 1.76), urban residents (HR 1.81), no-diabetes (1.67), and no-hypertension (1.87). The Fig 3 showed that the 95% CI between categories is overlapped because the HR for each category is represented by one picture. Each category of subgroup was analyzed independently and has an independent meaning.

## Discussion

AS patients have many comorbidities, including cardiovascular diseases and psychiatric disorders.[4,5,10,12–15] The present study specifically focused on dementia, which has been becoming a major issue both in the medical and socioeconomic fields.[16,17] Based on the results of a present study using nationwide, longitudinal cohort data, the prevalence of overall dementia (1.37%) and AD (0.99%) in the AS group was significantly higher than those of the control group (0.87% and 0.63%), respectively. Adjusted HR of the AS group for overall dementia and AD also showed statistical significance. On the other hand, the prevalence of VD was not significantly different between the two groups. There are a number of possible explanations for these results.

First, AS and dementia have multiple equal causes and medical comorbidities.[4,12] Norton presented seven risk factors for dementia in 2014: low educational level, obesity, hypertension, low physical activity, smoking, diabetes, depression. [18] As with these disorders, hypertension, diabetes, and dyslipidemia were known as chronic conditions that can induce both AS and dementia. In other words, AS and dementia might be associated with diseases, sharing multiple causative illnesses. According to numerous studies, chronic uncontrolled inflammation has been known to be a mechanism responsible for not only AS but also psychiatric disorders including dementia. Second, not only the medical diseases, but the morbidity of psychiatric disorder in AS is high, due to symptoms such as chronic pain and functional impairment, and the association with depression and anxiety has already been revealed.[5,8,10,12] Consequently, this poor psychological status alone could be a direct cause of AD. The third hypothesis is that the extracellular deposit of amyloid, which is known as one of the pathophysiologies of the AD, is implicated.[19] In the AD patient, apolipoprotein E4 inhibits serum amyloid excretion, promoting disease activity due to amyloid deposit. Similarly, AS patients show an increased level of serum amyloid.[20,21] Amyloid has been known as a useful indicator of disease activity, and it has shown a significant relationship with the markers (Bath Ankylosing Spondylitis Disease Activity Index score, erythrocyte sedimentation rate, and C-reactive protein) in AS.[20,21] The fourth hypothesis is related to the long-term persistent decline of physical activity in those with AS. Because enthesopathy involves the multiple skeletal organs and symptoms usually develop relatively early in life, the AS patient shows decreased physical activity from a young age and chronic progression of symptoms over time.[6–8,10] As a natural result, decreased social, physical, and workforce activity are common findings (work disability, unemployment, disturbed sexual function, decreased the quality of life, and fatigue symptoms).[6,7,10,11] Physical activity is known to have a significant effect on cognitive ability. These conditions may lead to chronic psychological distress, which might result in dementia in old age, as a long-term outcome.[8] This hypothesis could be persuasive due to the well-known fact that the amount of activity at a young age is related to the incidence of dementia. The fifth hypothesis is based on an advancement of the treatment and increased lifetime of AS patients. Newly developed drugs increase the survival period and rate; therefore, the number of elderly patients of advancing age with AS will grow, and this will be accompanied by a high incidence of dementia among them, the representative senile disorder. Our data also showed the time-dependent increase in the gap difference between two KM curves of the AS and control groups (Fig 2A and 2B).

It is noteworthy that the AS of our study did not show any significant correlation with VD, despite the higher incidence of cardiovascular problems in AS patients. However, the general cardiovascular disorder usually occurs in the large vessels, and the casual lesion that induces VD occurs in the small-sized vascular region of the brain.[22] Therefore, it is a natural phenomenon, considering that the general cardiovascular problems and VD are distinguished disease entities with different pathophysiology.

In subgroup analysis, male gender showed higher HR for dementia than female. We suggest a number of possible explanations for the result. First, the severity of AS including status of inflammation might be differed by gender. Indeed, male gender showed a higher incidence rate of disability and radiographic damage in AS patient. Because several studies suggested the relationship between the chronic inflammation and dementia, we also carefully considered the possible affection of severe AS (chronic inflammatory rheumatic disease) to the higher prevalence of dementia.[23, 24] Future study will need to have data or information about the status of AS inflammation. Second, we need to discuss the affection of the gender itself to dementia. Female gender has a higher prevalence of dementia in the general population. However, this result might due to the longer female life expectancy or sociocultural detection bias, rather, a few papers revealed the education level as a more significant risk factor than gender. Interestingly, a recent meta-analysis found that there is no significant difference in dementia development between male and female gender after controlling for sex differences in longevity.[25] In subgroup analysis, subjects older than 65 years also showed higher HR for dementia than other age groups. These results may be due to differences in the prevalence of dementia according to age group. The incidence of dementia in subjects younger than 40 years of age is too low that the AS probably could not increase the incidence of dementia with statistical significance. However, as the age increases, subjects with age 40–64 showed borderline significance (95% CI, 1.00–2.24) and the subjects with age greater than 65 having a higher prevalence of dementia showed statistical significance. In addition, subjects with dementia less than 65 years of age were known to a different type such as young-onset dementia having disparate characteristics distinguished from general type dementia commonly observed in elderly patients. Young-onset dementia is more likely to cause problems with movement, walking, coordination or balance and related to be hereditary. Authors suggest theses hypothesis as the reasons that each age group showed different HRs and AS significantly affected the incidence of dementia in the greater-than-65-year-old group.

Dementia is a major disease that increases the socio-economic burden on society. In Korea, each patient should pay an average of $18,000 annually for dementia management, and the total cost for dementia care is expected to rise rapidly to $20 billion in 2030 and $30 billion in 2040. Therefore, considering the severity of dementia, finding a high-risk group of vulnerable subjects based on various risk factors is a fundamental task in the prevention of disease in AS patients. In the present study, subgroup analyses revealed risk factors for dementia in the AS group: male gender, greater than 65 years group, fair income, urban residents, no-diabetes, and no-hypertension. Therefore, we can suggest that comprehensive care for preventing dementia is needed for AS patients having the aforementioned demographics and medical conditions. It could be another distinguished point of our study from other research that we evaluated various related factors including comorbid medical disorders (hypertension, diabetes, and dyslipidemia) as well as against large-scale data. We also reported the regression analysis after adjusting for possible confounding factors.

The present study has a few limitations. First, risk factor analysis for AS and dementia was not complete because there was a lack of assessment for detailed clinical variables in the K-NHIS dataset, such as smoking, alcohol consumption, body mass index, and family history of psychiatric illness. However, the present study showed several significant strengths compared to the other cross-sectional data, including large-sized sample, nationwide population-based dataset, and longitudinal cohort study design. Second, because the K-NHIS claim database doesn't include the detailed clinical results (laboratory and radiological findings or disease-specific scores), data or information about the status of AS inflammation did not evaluate in the present study. These shortcomings are known as inevitable limitations of the National Health Insurance Claim dataset. Third, the diagnoses of AS are identified using the ICD-10-CM codes, and its prevalence may be underestimated because only subjects seeking a medical evaluation can be identified; however, this would most likely result in an underestimate of the association between AS and psychiatric disorders. Fourth, there may be a shortage of evaluating psychological status related to dementia. Although the depression variable was included and analyzed in the present study, they did not include variables such as anxiety and sleep abnormality due to the practical limitations of researchers.

## Conclusion

The results of the present study, a nationwide, population-based, retrospective, longitudinal cohort study, showed that AS patients have a significantly higher prevalence of overall dementia and Alzheimer’s dementia than the general population. Comprehensive patient assessment using our subgroup analysis could help to prevent dementia in patients suffering from AS.
